# Mitochondrial Myopathy, Encephalopathy, Lactic acidosis and Stroke-Like Episodes Syndrome Presenting With Anton-Babinski Syndrome and Concurrent Occipital Lobe Seizures

**DOI:** 10.7759/cureus.12908

**Published:** 2021-01-25

**Authors:** Amr Ewida, Rashid Ahmed, Anqi Luo, Hesham T Ghonim, Arayamparambil C Anilkumar

**Affiliations:** 1 Neurology, Upstate University Hospital, Syracuse, USA; 2 Neurology, University of Texas (UT) Health Science Center at San Antonio, San Antonio, USA; 3 Neurology, University of Pittsburgh Medical Center, Pittsburgh, USA

**Keywords:** melas syndrome, occipital seizures, cortical blindness, anton–babinski syndrome, anton syndrome

## Abstract

Mitochondrial encephalomyopathy, lactic acidosis, and stroke-like episodes (MELAS) is a complex group of disorders with multisystem involvement that have a wide range of biochemical and genetic defects. The earliest symptoms of MELAS typically include easy fatigability, muscle weakness, encephalopathy with stroke-like episodes, recurrent headaches and seizures. The pathogenesis of stroke-like episodes manifesting as focal deficits like acute cortical blindness is not fully understood. We present an eight-year-old, right-handed boy with MELAS confirmed by the presence of pathogenic missense variant mutation (mt.3243A>G) presenting with acute intermittent reversible episodes of cortical blindness and Anton-Babinski Syndrome secondary to concurrent occipital lobe seizures captured during video electroencephalography (V-EEG) monitoring, in addition to the neuro-imaging which was not consistent with acute ischemic stroke.

This case highlights the importance of the V-EEG monitoring besides clinical testing and radiographic correlation during acute cortical blindness episodes in MELAS as occipital lobe seizures could be a part of the symptomatology.

## Introduction

The syndrome of mitochondrial myopathy, encephalopathy, lactic acidosis and stroke-like episodes (MELAS; OMIM #540000) is a maternally inherited disorder due to mitochondrial deoxyribonucleic acid (DNA) point mutation. Epilepsy is a common manifestation of MELAS. Symptomatic occipital lobe epilepsy and acute cortical blindness are increasingly recognized. Nevertheless, the pathogenesis of acute cortical blindness in MELAS patients is not fully understood and mostly considered related to the stroke-like episodes [1]. Cortical blindness with Anton-Babinski syndrome is a rare condition caused by multiple etiologies with only 28 cases reported between 1965 and 2016 [2]. To the best of our knowledge, there is only one case report about MELAS syndrome presenting with Anton-Babinski Syndrome secondary to stroke-like episodes [3].

We report an eight-year-old boy who presented with a stroke-like episode, followed by intermittent acute cortical blindness and Anton-Babinski Syndrome attributable to concomitant occipital lobe seizures with clinical, radiological and electroencephalographic evidence.

## Case presentation

**Figure 1:**
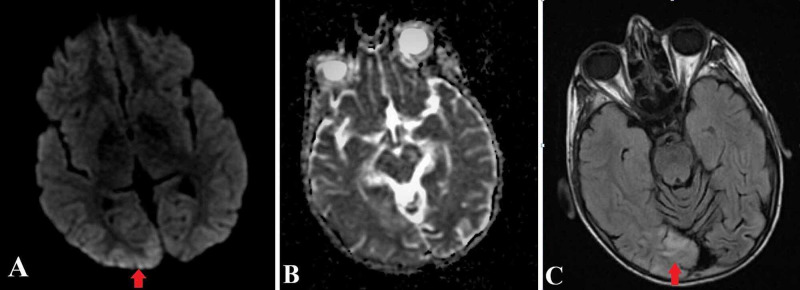
MRI sequences show restricted diffusion on DWI (A) in the right occipital region which is isodense on ADC (B) and hyperintense on T2-FLAIR (C), respectively. DWI: diffusion-weighted imaging, ADC: apparent diffusion coefficient, T2-FLAIR: T2 fluid inverted attenuation recovery.

**Figure 2:**
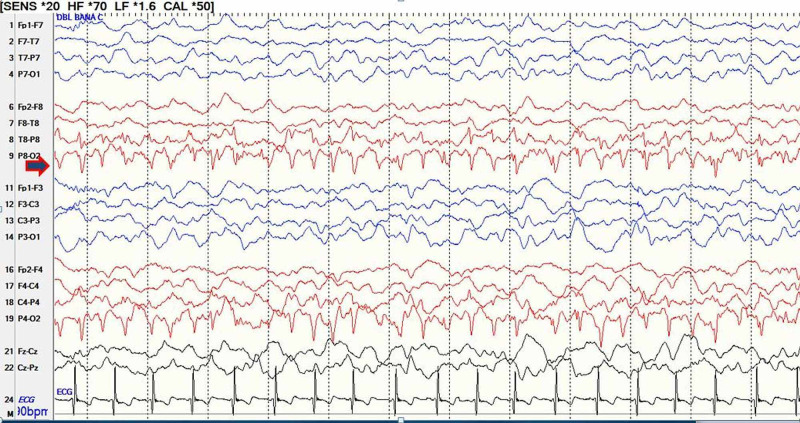
Scalp EEG shows evolving right occipital seizure before spread which is concomitant with transient cortical blindness episodes. EEG: electroencephalogram.

An eight-year-old, right-handed boy with recently diagnosed MELAS presented with sudden onset of vomiting, vision changes, fevers, and malaise, followed by intermittent episodes of acute cortical blindness. The fundus examination was normal with intact pupillary reflexes and visual acuity. During these episodes, the patient was also noted to have Anton-Babinski syndrome with the inability to discern objects, color or people around him with anosognosia and confabulation. Video electroencephalography (V-EEG) monitoring during these episodes revealed multiple occipital seizures emerging from the right occipital lobe with rapid spread to the left occipital region (Figure 1). These electrographic seizures correlated with the episodes of cortical blindness and the patient intermittently regained his vision with the absence of the ictal pattern. Magnetic resonance imaging (MRI) of the brain with and without contrast showed abnormal T2 fluid inverted attenuation recovery (FLAIR) hyperintensity of the cortex and subcortical white matter of the occipital lobes, greater on the right with corresponding mild cortical restricted diffusion with no drop out signals on apparent diffusion coefficient (ADC) sequence (Figure 2). The constellations of findings were related to postictal changes. His condition improved with optimizing anti-seizure medications, by administering lorazepam, adjusting the levetiracetam dose and adding lacosamide as a second drug. In addition, he was discharged on L-Arginine, levocarnitine, supplements of vitamins: C, B1, B2 and B12.

## Discussion

Although all mitochondrial disorders (MDs) constitute a heterogeneous group of diseases that can affect virtually any organs and system, they have a strong predilection toward the central nervous system (CNS). Among the broad spectrum of CNS manifestations, a seizure is one of the most common symptoms, and the reported seizure frequency in children with MDs ranges from 35% to 61% [4]. While it is known that MELAS has a tendency to affect mainly the posterior quadrants of the brain and can cause cortical blindness and occipital lobe seizures [5]. However, the exact pathogenesis of stroke-like episodes and subsequent acute cortical blindness is not clearly understood. Whether acute cortical blindness in MELAS is directly linked to mitochondrial-related cortical dysfunction or succeeding ictal phenomena or both, is uncertain at this time.

In general, occipital lobe seizures develop within seconds, last less than two to three minutes and consist of simple visual hallucination phenomena of multicolored and circular patterns with or without ictal blindness [6]. The continuous V-EEG done in our case during the cortical blindness episodes supported the diagnosis of focal seizure, originating from the right occipital lobe region and then propagates to the left occipital region. The right occipital lobe showed the area of DWI and FLAIR sequence hyperintensity. A summary of related literature on EEG findings and seizure types in mitochondrial disorders are shown in Table 1 [3,4,7-10]. This highlights the fact that our patient is a rare MELAS case of acute cortical blindness episode with Anton-Babinski syndrome due to occipital lobe seizures with clinical, radiological and electroencephalographic concordance (Figures 1 and 2).

**Table 1:** Summary of seizure types and EEG findings in mitochondrial disorders from related literature. MELAS: mitochondrial myopathy, encephalopathy, lactic acidosis and stroke-like episodes, EEG: electroencephalogram.

Study	Numbers	EEG findings	Seizures semiology and other comments
Alemdar et al. [3]	1	Diffuse slowing bilaterally without any epileptiform discharge.	Generalized tonic-clonic seizures and Anton-Babinski syndrome.
Chevallier et al. [4]	109	Generalized slowing (60%), multifocal discharges (41%), focal discharges (39%), generalized discharges (39%).	The most common seizure type in MELAS was a generalized tonic-clonic type (33%), with generalized or focal discharges.
Serra et al. [7]	12	Slow background activity; focal or generalized delta-theta activity or delta sharp waves; generalized sharp waves with spike/polyspike and slow-wave complexes activated by photic stimulation.	Myoclonic jerks, generalized seizures.
Demaret et al. [8]	7	Most presented with infrequent prolonged focal seizures and/or status epilepticus; periodic lateralized epileptiform discharges.	Epilepsia partialis continua, hemiclonic status epilepticus, non-convulsive status, and occipital status epilepticus. Seizures usually occurred during the acute phase of stroke-like episodes.
Fujimoto et al. [9]	6	Focal high-voltage delta waves with polyspikes, which were recognized as an ictal EEG pattern. In subacute and chronic stages, focal spikes or sharp waves and 14- and 6-Hz positive bursts.	Focal clonic or myoclonic seizure and migrainous headache.
Abboud and Sabbagh [10]	1	Diffuse sharp waves with focal epileptic discharges over the posterior head region.	Acute blindness, generalized tonic-clonic seizures and occipital headache with nausea and vomiting.

Like other studies, our patient’s occipital epilepsy has a temporal relationship with his stroke-like episode. Our patient presented initially with fatigue, temporary focal weakness. Only transient cortical blindness manifested as Anton-Babinski syndrome was associated with concurrent electrographic changes of occipital seizures. Stroke-like episodes are usually manifested by a mixture of features including altered consciousness, seizures, and transient focal deficits, without restriction to cerebrovascular territories [1,5]. Some authors have provided pathological evidence that there are capillary proliferation and increased numbers of abnormal mitochondria in both endothelial and smooth muscle cells in the small arterioles of the brain [11,12]. A seizure can lead to rapid energy depletion in the neurovascular units composed of brain endothelial cells, pericytes, astrocytes and neurons. The energy depletion in these neurovascular units can produce post-ictal Todd’s paralysis. Therefore, Fryer et al. proposed that the triggering event in the stroke-like episode is possibly a seizure, which would explain well the association between the stroke-like episode and the seizure in our patient [1,8]. MELAS often presents with either complex partial or generalized seizures and frequently with status epilepticus [13]. The symptomatology of occipital lobe epilepsy was first described by Gowers in 1885 who reported patients with visual warnings consisting of elementary hallucinations and amaurosis [14]. Occipital lobe seizures manifesting as positive visual phenomena, such as hallucinations in addition to blindness which is a common ictal or postictal symptom of idiopathic and symptomatic occipital epilepsy, but sometimes ictal and postictal blindness cannot be clearly differentiated. Reasons include the inherent limitations of scalp EEG recordings from the occipital region and the fact that symptomatology may be secondary to ictal spread rather than to the origin of the epileptogenic discharge [13,15]. Therefore, it is not easy to capture the perfect clinical-electrographic correlation in occipital lobe epilepsy.

Neuroimaging in our patient revealed hyperintensity on DWI as well as FLAIR and T2 signal along the right occipital cortex, but the corresponding occipital region had iso-density in ADC values, which rules out restricted water diffusion as in an ischemic stroke. This temporal ADC evolution in status epilepticus contrasts with the pattern seen in ischemic stroke, where ADC value is significantly reduced in the acute phase, followed by a gradual increase in the following days [16]. The local cellular disturbance initiated by a seizure may lead to fluctuations and shift of intra- and extra-cellular water, possibly resulting in cell swelling. These changes in the water environment usually occur as an increase in T2-weight signal intensity [17]. The hyperintensity of DWI observed in the acute phase of seizure-induced lesions and ischemic stroke can be similar. DWI abnormalities in seizures result from a combination of impaired energy metabolism and hemodynamic changes rather than hypoxia/anoxia present in acute ischemic stroke. Therefore, the DWI in seizures frequently shows reversibility [16].

## Conclusions

Mitochondrial disorders are important differential diagnostic considerations in pediatric occipital lobe epilepsy. Our case adds to the limited literature on understanding the pathogenesis of acute cortical blindness episodes in MELAS with occipital lobe epilepsy which could be a culprit in symptomatology. In our case, acute intermittent cortical blindness episodes presented as Anton-Babinski syndrome and were associated with simultaneous occipital lobe seizures as evidenced by V-EEG monitoring and MRI findings. V-EEG monitoring in these patients seems to be an important tool for evaluation as manifestations of occipital lobe seizures could be subtle and at times, non-specific which can be clinically challenging in the pediatric population.

## References

[1] Fryer RH, Bain JM, De Vivo DC (2016). Mitochondrial encephalomyopathy lactic acidosis and stroke-like episodes (MELAS): a case report and critical reappraisal of treatment options. Pediatr Neurol.

[2] Kim N, Anbarasan D, Howard J (2017). Anton syndrome as a result of MS exacerbation. Neurol Clin Pract.

[3] Alemdar M, Iseri P, Selekler M, Budak F, Demirci A, Komsuoglu SS (2007). MELAS presented with status epilepticus and Anton-Babinski syndrome; value of ADC mapping in MELAS. J Neuropsychiatry Clin Neurosci.

[4] Chevallier JA, Von Allmen GK, Koenig MK (2014). Seizure semiology and EEG findings in mitochondrial diseases. Epilepsia.

[5] Pavlakis SG, Phillips PC, DiMauro S, De Vivo DC, Rowland LP (1984). Mitochondrial myopathy, encephalopathy, lactic acidosis, and strokelike episodes: a distinctive clinical syndrome. Ann Neurol.

[6] Caraballo R, Koutroumanidis M, Panayiotopoulos CP, Fejerman N (2009). Idiopathic childhood occipital epilepsy of Gastaut: a review and differentiation from migraine and other epilepsies. J Child Neurol.

[7] Serra G, Piccinnu R, Tondi M, Muntoni F, Zeviani M, Mastropaolo C (1996). Clinical and EEG findings in eleven patients affected by mitochondrial encephalomyopathy with MERRF-MELAS overlap. Brain Dev.

[8] Demarest ST, Whitehead MT, Turnacioglu S, Pearl PL, Gropman AL (2014). Phenotypic analysis of epilepsy in the mitochondrial encephalomyopathy, lactic acidosis, and strokelike episodes-associated mitochondrial DNA A3243G mutation. J Child Neurol.

[9] Fujimoto S, Mizuno K, Shibata H (1999). Serial electroencephalographic findings in patients with MELAS. Pediatr Neurol.

[10] Abboud H, Sabbagh C (2008). Acute blindness. Emerg Med J.

[11] Betts J, Jaros E, Perry RH (2006). Molecular neuropathology of MELAS: level of heteroplasmy in individual neurones and evidence of extensive vascular involvement. Neuropathol Appl Neurobiol.

[12] Sparaco M, Bonilla E, DiMauro S, Powers JM (1993). Neuropathology of mitochondrial encephalomyopathies due to mitochondrial DNA defects. J Neuropathol Exp Neurol.

[13] Kaufman KR, Zuber N, Rueda-Lara MA, Tobia A (2010). MELAS with recurrent complex partial seizures, nonconvulsive status epilepticus, psychosis, and behavioral disturbances: case analysis with literature review. Epilepsy Behav.

[14] Gowers WR (1885). Epilepsy and other chronic convulsive disorders. New York : William Wood & Company.

[15] Kuzniecky R (1998). Symptomatic occipital lobe epilepsy. Epilepsia.

[16] Shaw S, Kim P, Millett D (2012). Status epilepticus amauroticus revisited: ictal and peri-ictal homonymous hemianopsia. Arch Neurol.

[17] Kim JA, Chung JI, Yoon PH, Kim DI, Chung TS, Kim EJ, Jeong EK (2001). Transient MR signal changes in patients with generalized tonicoclonic seizure or status epilepticus: periictal diffusion-weighted imaging. AJNR Am J Neuroradiol.

